# Supplementary material for: Stacking Model‐Based Classifiers for Dealing With Multiple Sets of Noisy Labels
Source: Biom J. 2025 Mar 12;67(2):e70042. doi: 10.1002/bimj.70042 (PMC11898607; doi:10.1002/bimj.70042)
Supplement: Supplementary file 1 — Supporting Information [file BIMJ-67-e70042-s001.zip › density-based-ensemble-model-main/competitors_real_data/netlab-master/netlab-master/help/mlphint.htm]

Netlab Reference Manual mlphint


# mlphint

## Purpose

Plot Hinton diagram for 2-layer feed-forward network.

## Synopsis

```
mlphint(net)
[h1, h2] = mlphint(net)
```

## Description

`mlphint(net)` takes a network structure `net`
and plots the Hinton diagram comprised of two
figure windows, one displaying the first-layer weights and biases, and
one displaying the second-layer weights and biases.

`[h1, h2] = mlphint(net)` also returns handles `h1` and
`h2` to the figures which can be used, for instance, to delete the
figures when they are no longer needed.

To print the figure correctly, you should call
`set(h, 'InvertHardCopy', 'on')` before printing.

## See Also

`demhint`, `hintmat`, `mlp`, `mlppak`, `mlpunpak`

---

**Pages:**
Index


---

Copyright (c) Ian T Nabney (1996-9)
